# Family physicians’ experience and understanding of evidence-based practice and guideline implementation in primary care practice, Cape Town, South Africa

**DOI:** 10.4102/phcfm.v11i1.1592

**Published:** 2019-05-27

**Authors:** Michael K. Pather, Robert Mash

**Affiliations:** 1 Division of Family Medicine and Primary Care, Faculty of Medicine and Health Sciences, Stellenbosch University, Cape Town, South Africa

**Keywords:** evidence-based practice, guideline implementation, primary health care practice

## Abstract

**Background:**

In primary care, patients present with multimorbidity and a wide spectrum of undifferentiated illnesses, which makes the application of evidence-based practice (EBP) principles more challenging than in other practice contexts.

**Aim:**

The goal of this study was to explore the experiences and understanding of family physicians (FP) in primary care with regard to EBP and the implementation of evidence-based guidelines.

**Setting:**

The study was conducted in Cape Town primary care facilities and South African university departments of Family Medicine.

**Methods:**

For this phenomenological, qualitative study, 27 purposefully selected FPs from three groups were interviewed: senior academic FPs; local FPs in public-sector practice; and local FPs in private-sector practice. Data were analysed using the framework method with the assistance of ATLAS.ti, version 6.1.

**Results:**

Guideline development should be a more inclusive process that incorporates more evidence from primary care. Contextualisation should happen at an organisational level and may include adaptation as well as the development of practical or integrated tools. Organisations should ensure synergy between corporate and clinical governance activities. Dissemination should ensure that all practitioners are aware of and know how to access guidelines. Implementation should include training that is interactive and recognises individual practitioners’ readiness to change, as well as local barriers. Quality improvement cycles may reinforce implementation and provide feedback on the process.

**Conclusion:**

Evidence-based practice is currently limited in its capacity to inform primary care. The conceptual framework provided illustrates the key steps in guideline development, contextualisation, dissemination, implementation and evaluation, as well as the interconnections between steps and barriers or enablers to progress. The framework may be useful for policymakers, health care managers and practitioners in similar settings.

## Introduction

One of the most consistent findings in health services research is the gap between evidence and practice.^1,2,3^ Bridging this gap is central to ensuring that beneficial interventions are used appropriately and harmful interventions are avoided.^4^

The principles of evidence-based practice (EBP), however, are not as easily applied in primary care as in the biomedical and more disease-centred context of hospital care, where the process of evidence-based medicine (EBM) was originally developed.^5^ Primary care is characterised by undifferentiated multiple problems across a broad spectrum of reasons for encounter with a complex interplay of medical, social and psychological issues. Practitioners need research evidence for the whole range of diagnostic, prognostic, interventional and phenomenological questions that arise within this context.^6^ Concern has also been raised that research is often conducted in areas outside of primary care with limited generalisability.^7^ In addition research evidence from the primary care setting is often of poor quality^8^ and evidence derived from randomised controlled trials constitutes only a portion of the real knowledge that is needed.^9^

Complexity theory has influenced the philosophical discourse of family medicine and provides tools to understand the consultation.^10^ Primary health care (PHC) is complex and uncertainty is common.^11^ In addition, knowledge of complexity is critical in the management of patients with comorbidity in PHC practice^12^ and makes both research and delivery of care particularly challenging.^13^

One of the most common ways of synthesising and presenting the latest evidence to clinicians is in the form of clinical practice guidelines. As EBP grew, more and more guidelines were prepared and disseminated, many for use in primary care. Clinical practice guidelines respond to wide variations in practice, excessive cost, substandard outcomes and new evidence, which could have a significant impact on patient management.^14^ Clinical practice guidelines, however, struggle to deal with complexity and to guide decision-making in the face of comorbidity.

Clinical practice guidelines have been shown to change clinical practice and improve patient outcomes.^15,16^ Although a quality evidence-based guideline has the potential to achieve this, it may only succeed if ‘as much attention is paid to the dissemination and implementation phase as to its original development’.^17^

Poor dissemination of guidelines results in poor availability at the point of care and therefore little impact on health care delivery.^18,19^ In developing countries up to 60% of guidelines that are available centrally may be absent in more peripheral settings.^20^

Guideline implementation is an active, planned and tailored process, which addresses barriers to change.^21^ Even in well-resourced settings implementation may be patchy, and large variations in practice remain.^22,23^ It is not clear whether guideline developers, managers of health organisations or practitioners should be responsible for implementation, and implementation is often poorly funded.^24^

Understanding the knowledge, attitudes and perspectives of practitioners can improve their adherence to guideline recommendations^25^ and more could be done to adapt guidelines to local settings.^26^ Engaging the users of the guidelines in implementation has been recommended,^27^ but the most effective strategies for implementation remain unknown. Multifaceted interventions were thought to be more effective, but recent evidence suggests otherwise.^28,29^ Implementation should go beyond staff education to look at the organisation of care and teamwork. Staff education should embrace different learning styles and principles of adult education.^30^

Enablers of guideline implementation include feedback, educational outreach, face-to-face training of practitioners by professional experts and quality improvement cycles.^31,32,33^ Educational outreach or ‘academic detailing’ in primary care has been shown to be effective in the local context.^30,34,35,36^ Barriers to guideline implementation include time constraints, lack of motivation and clinical inertia,^37,38^ inappropriate evidence, the capability and preferences of the health care providers, and the capacity of the health care setting.^39^

The integration of evidence-based decision-making with the complexity of primary care may require additional tools such as using an empirical trial of treatment^40^; decision-making on the basis of experience, evidence and knowledge of the patient’s story^41^; goal-orientated care^42^; plan–do–study–act cycles^43^; and using problem-solving techniques.^44^ Effective clinical decision-making therefore requires a holistic approach that accepts unpredictability and uncertainty, good communication skills; and good judgment to balance competing interests.^45^

In South Africa, little is known about the attitudes and behaviour of primary care practitioners towards EBP and the implementation of clinical practice guidelines. This research aimed to explore the experiences and understanding of family physicians (FPs) in South African primary care with regard to EBP and the implementation of evidence-based guidelines in practice.

## Methods

### Design

This was a phenomenological, qualitative study using semi-structured interviews.^46^

### Setting

In the public sector of the Cape Town metropole, FPs were appointed at community health centres where they were the senior clinicians in multidisciplinary teams that included medical officers, clinical nurse practitioners, pharmacists and other allied health professionals. Their job description included leadership of clinical governance, and one of the expected activities was implementation of clinical guidelines. In the private sector, FPs were mostly in solo practice. Some of the FPs were also involved with the clinical training of under- or postgraduate students and were linked to universities. Public sector facilities served the majority of the uninsured population, while the private sector served those with insurance or able to make out-of-pocket payments. Family physicians were trained by academic departments at eight different universities in South Africa and these departments employed full-time academic FPs at the level of senior lecturer or professor. In Cape Town, Stellenbosch University and the University of Cape Town were training FPs.

### Selection of family physicians

Purposeful sampling was used to select 8–12 FPs from three different groups: senior academic FPs affiliated to universities in South Africa; local FPs in the public sector; and local FPs in private practice. All the local FPs selected had some involvement with the universities in the Cape Town metropole as this would ensure some exposure to EBP and guideline implementation. Recruitment procedures also drew upon snowball sampling, as interviewees suggested other FPs who had the capacity to contribute to the phenomenon of interest.

### Data collection

All interviews were conducted face-to-face and were digitally recorded by the researcher in the interviewee’s workplace. One interview was conducted in Afrikaans and all other interviews in English. An interview guide consisting of broad open questions and topics for exploration was used to generate discussion rather than to elicit answers to specific questions. Features of the particular context (the health centre or private practice) were explored. This included the attitudes of colleagues in the public and private sector towards EBP and guideline implementation, the style of leadership in their practices as well as features of the strategies used for dissemination and implementation of guidelines. Other areas explored were the concept of evidence; FPs’ views of the barriers to guideline implementation; their views on how best to implement guidelines; anecdotal experience in practice and the role of the patient in clinical decision-making.

### Data analysis

All interviews were transcribed verbatim and were returned to the interviewees for member checking. Analysis used the framework method,^47,48^ which involved the following steps:

**Familiarisation:** The researcher immersed himself in the transcripts by reading, rereading and listening to the original recorded interviews and referring to field notes taken during the interviews in order to become familiar with the data.

**Identification of a thematic index:** The researcher created a list of codes and organised them into categories based on a detailed analysis of a sample of the transcripts. Codes were identified inductively from the data and organised in relation to the aim and objectives of the study.

**Indexing:** The transcripts were systematically coded using the thematic index and with the assistance of a qualitative data analysis software program, ATLAS.ti, version 6.1.^49^

**Charting:** Codes related to a particular category (family as designated by ATLAS.ti) were then collated together in ATLAS.ti and the output saved or printed.

**Interpretation:** The charts were used to interpret the data and identify emerging themes. Connections between different themes, the range and strengths of different opinions within themes as well as contradictions were reflected on. The researcher searched for alternative explanations and potential negative cases. A reflexive report was referred to in an attempt to remain neutral and receptive to the data during this phase of the analysis. The reflexive notes dealt with any assumptions, predispositions, biases and perspectives of the researcher with regard to EBP and guideline implementation.

### Reflexivity

In the following two paragraphs, I provide a very brief summary of self in relation to the research conducted, in an attempt to reflexively outline the potential predispositions that could have influenced the way the research was planned, conducted, analysed and interpreted.

I worked in the PHC sector of the Cape Town metropole for a period of 20 years consecutively from 1986 to 2006 and rotated through many of the Community Health Centres (CHCs) in the Cape Town metropole. During this time I became well aware of the scope of practice and areas of potential weakness in the health system. Weaknesses noted and that prompted this research included the wide variation in practice; opinion based practice (especially of the older generation of practitioners); a paucity of implemented evidence-based guidelines to assist health care workers, particularly in the management of diseases of chronic lifestyle; the inability to incorporate current evidence in decision-making (even where evidence-based guidelines were available); the generally poor quality of care as perceived by patients and medical staff; and the lack of continuity of care coupled with the ongoing frustrations of an ever-increasing workload within a resource-constrained context of practice. It was clear that no formal implementation process for guidelines existed and practitioners were in most instances not even aware of available guidelines.

I pursued postgraduate studies and completed a master’s degree in Family Medicine and Primary Care at Stellenbosch University in December 1995 and returned to practice in the same metro district health systems as a senior family physician for a period of 10 years. I was also appointed as a full-time senior lecturer in the Division of Family Medicine and Primary Care, Stellenbosch University, in January 2007 and was mainly involved in the formal training of undergraduate and postgraduate students in EBP during family medicine training attachments. As a proponent of EBP, my main focus of teaching was EBP at the point of care, and I taught the EBP process, searching for clinical research evidence, critical appraisal and the application of evidence in clinical decision-making. Therefore, my background knowledge of EBP could have impacted on the qualitative analysis performed.

### Ethical considerations

Permission was granted by the Health Research Ethics Committee of Stellenbosch University (project number: N07/03/066) and permission was obtained from the Metro District Health Systems of the Western Cape to conduct this research study.

## Results

A total of 27 FPs were interviewed from three groups: 10 were academic FPs affiliated to departments of family medicine nationally, 10 were FPs in the Cape Town public sector and 7 were FPs from the private sector.

### Evidence quality and relevance

Family physicians felt that research evidence forms an important component of decision-making in clinical practice. However, rigorously prepared quality evidence, derived from contexts other than primary care, might not be directly applicable and could be limited in its ability to influence practice. On the other hand, badly conducted research from the primary care setting might be equally unhelpful:

‘Well, I think for me, the best evidence would be evidence that comes from research done in the context where I work, and not necessarily done in a setting that’s not relevant to where I work.’ (FP9)

To deal with patient problems effectively required an awareness of the whole person and their illness experience with issues such as patient values, expectations, beliefs and concerns in addition to considerations of clinical evidence and cost-effectiveness. It was clear that FPs recognised the limitations of EBM in addressing the problems with which patients present and the need for different forms of research evidence to inform their practice:

‘Evidence-based medicine, because we’re using a scientific method, it’s mainly looking at the biomedical side of issues. It doesn’t delve much into the psychosocial, cultural, political and administrative issues involved in patient care. So, in other words it’s limited.’ (FP3)

Family physicians felt that guideline recommendations needed to be more relevant to the inherent uncertainties, multimorbidity and undifferentiated nature of primary care:

‘In primary healthcare, which is my home, we are riddled with a lot of uncertainties, and that is the nature of our discipline. So, it is particularly important to be able to access evidence for the kind of problems that we encounter. It is critical for us that we have some evidence for what we do, especially because most of what we see doesn’t really have state-of-the-art evidence, but we always do our best to access whatever evidence there is around us.’ (FP1)

Family physicians had some ambivalence and uncertainty about the potential for guidelines to improve health outcomes as they recognised that these outcomes were influenced by multiple factors and that evidence generated for a study population might not always translate to an effect for individual patients:

‘It is difficult to say that it will definitely lead to improved health outcomes. That is difficult to say. I would expect it would lead to improved health outcomes, but I won’t say definitely and promise someone, even a patient, that it will definitely.’ (FP7)

### Guideline development

Family physicians felt that guideline development teams should include representatives of district health services, primary care and patients, and not only so-called experts. All members of such an inclusive group have different types of expertise on what is relevant and useful evidence. Respondents felt that primary care practitioners should become more involved in providing the relevant research required to address the gaps in their knowledge and assist in the formal development of the guideline. However, this would be extremely challenging given the current workload they have to deal with:

‘Although in my experience in our country, I have found that most guidelines are developed by subspecialists without involvement of primary care providers. That for me creates an impression that the specialists, or subspecialists, are relied upon to give direction to primary care providers. Not that I have a problem with that, but I feel that primary care providers must also be involved in the drawing up of these guidelines. But then that also means that primary healthcare providers must also be involved in research activities, because to be able to say to people, ‘this is what we experience in primary healthcare’, one should be doing research in one’s environment.’ (FP2)

Family physicians felt that qualitative research was underutilised as this form of research could provide unique contextual insight into important areas such as how to improve adherence of patients to medication, or how to get primary care practitioners to change their behaviour in clinical practice:

‘We are very good at doing qualitative work. We bring it on board and say these are the reasons why our patients are struggling with adherence. These are the reasons why our patients are not able to keep up with lifestyle modifications. In that way, the guideline will come out much, much richer and much, much more relevant to our context.’ (FP3)

Although FPs felt that guideline development should be more inclusive, they also thought that the underlying synthesis and critical appraisal of the research evidence should be a national process led by South African universities:

‘So, my personal feeling is even [*a*] national protocol or national guideline, once it is made, it would be nice for especially the academic departments to take it up and look at the evidence on which this was made …’ (FP12)

Once the guideline content has been finalised the structure and layout is important to facilitate the usability of the guidelines by the target audience. Guidelines should be concise and easy to use:

‘You can’t write guidelines that makes provision for every individual variety. Then the guidelines will be thick books, and you want the guideline to be short and simple and generic, that it can be applied in a lot of different circumstances to make it easy, because if a guideline is also too long-winded, people won’t read it.’ (FP14)

Guidelines should be easily accessible:

‘So, there must be easy access to the guidelines, and also appropriate marketing of the guidelines. If there is easy access, there may be better utilisation. Now, for that purpose, your practitioners who are going to be using guidelines need to then develop the skill to access material easily and quickly.’ (FP5)

Guidelines should have a more standard structure and way of presenting recommendations and there should not be multiple guidelines on the same topic at the point of use. Family physicians may need to help select the most appropriate guidelines or recommendations:

‘In our setting we don’t have a structured way of the guidelines being presented. There are guidelines coming from the Department of Health and from other sources … There are the national guidelines which come out with the SAMJ [*South African Medical Journal*] … So, we have various sources of guidelines, and then our referral hospital, which is Paarl Hospital, also have their own set of local guidelines, from which they want us to prescribe. So, there’s not really uniformity or a structured way that we can decide this is the one we should stick to.’ (FP15)

### Contextualisation of the guideline

Adapting the guideline for local use was fundamental to the implementation process. This could include contextualisation to the user (i.e. scope of practice of clinical nurse practitioner, primary care doctor, family physician) and to the level of the health system (i.e. clinic, health centre, district hospital). Ownership of the adapted guideline should be at the level of the team, although ultimately individual practitioners will need to change their practice and may have different readiness to change:

‘The main thing about guidelines of course is that they must also be contextualised. There has to be that room for contextualisation of guidelines. That means that in a particular practice environment, the practitioners in that practice should be allowed to look at the guideline and so to speak, adapt it to their environment. But when they do that, they must do it as a team and they must explain why they are adapting the guideline, and then it must be accepted practice for that facility, and everybody must adhere to that modified guideline. It shouldn’t be an individual’s prerogative to modify guidelines as they go.’ (FP19)

Respondents in private practice highlighted the need for medical aid schemes to work with universities in an attempt to contextualise or adapt the recommendations to the cost constraints of managed health care:

‘I think if we can get guidelines that are set by academics in consultation with medical aids, I think that would be a start because then they could marry the evidence as well as the financial constraints that medical aids seem to find themselves in. So that would work well in private practice, and I think perhaps a different model, or a different set of guidelines should be set for patients who can’t afford medical aid, or who are not on medical aid. In other words, sort of state-based patients, which we would call ‘private patients’ in our practices.’ (FP13)

### Guideline dissemination

The processes of dissemination and implementation were seen as inter-related and should be handled as such. Respondents felt that great care must be taken when guidelines are disseminated in order to ensure that guidelines actually reach the target audience and are at least read. Awareness of new guidelines and their value was seen as a fundamental starting point:

‘I think the first thing is awareness. I am sometimes startled at how few practitioners are even aware of, or understand, what evidence-based medicine is, and guidelines are meant to achieve. So I think there is a level of awareness that has to be created.’ (FP2)

Dissemination, however, should not just mean sending it out but should also be linked to motivational strategies to stress the importance of the guideline, improve the confidence of recipients to use it and assist with the understanding of it. Such essential motivational steps improve readiness to use the guideline in practice and are crucial in the overall success of the dissemination process:

‘Well, how to do it, is not just to circulate it, but it needs to be discussed at facility level. So there needs to be someone who knows what goes on in the guideline and to either have been trained somewhere or to have read it properly, and then at facility level, it needs to be discussed by the clinicians. And then it helps if one has visual reminders in the room, or the manual in the room or whatever, that one can refer to when you’re not sure what to do with a patient.’ (FP10)

### Guideline implementation

Family physicians should lead by example in implementing the adapted guideline and should also include the key recommendations in quality improvement activities to check on implementation progress and achievement of health outcomes. This could also enable reflection on the guideline and an iterative process of checking if the guideline has been of value to practitioners and patients. A collaborative process of working through and prioritising the guideline recommendations in the local context with the clinicians was more likely to lead to implementation. The promotion of guidelines by external people or even local managers might be misinterpreted, for example, as being about cost-saving rather than quality of care:

‘So the way to do it is to get the guideline, give some in-service training, show by example how it’s used, implement it yourself so that people can see that you are doing it and you believe in it, and then also implement an audit system to check whether it improves the outcomes, certain specific outcomes.’ (FP16)

Others questioned who had the responsibility for implementation:

‘I don’t know if it’s the responsibility of the management or those who actually develop the guidelines to come through and make sure that there’s ongoing training, and ensuring that the people that you want to implement the guidelines understand and have the necessary knowledge to implement them properly.’ (FP17)

Time constraints, the lack of financial resources and how the health care system is organised were identified as major barriers to guideline implementation in the already overburdened primary care setting:

‘I think there are various reasons for that [*not implementing guidelines*]. From the simplest being that sometimes what is being proposed in the guideline takes more time, and often in government facilities you are pressed for time. So if you would make it relevant to asthma, the guideline says that you must check inhaler techniques, you must check the person’s understanding of the use of medication, and the junior medical officer, instead of following those steps, would just write up the medication to have a quick consultation.’ (FP17)

Respondents felt that practitioners must feel confident about the usefulness of their guideline. This means having a sense of cognitive resonance, the feeling of a positive emotional response and confidence that what is being recommended for patients is useful and effective:

‘So for me it’s about having that comfort, a lack of dissonance, the freedom of anxiety in prescribing a treatment plan, prescribing a medication or a system of treatment for that patient, knowing that it comes with tangible proof that it’s effective and that it’s working.’ (FP1)

Some FPs stressed the importance of all practitioner groups within the organisation being consistent in following the same guidelines. Such consistency may be difficult to achieve, especially as the development paths of clinical nurse practitioners (CNPs) and doctors are separate. In addition, tension exists between standardisation of care on the one hand and patients’ individual requirements on the other.

Formal and ongoing training on the use of a new guideline was essential to successful implementation:

‘Now, one thing about guidelines, I find if I have had some training, some form of a workshop in the line of a guideline, my chances of using and adhering to that guideline is much, much better, than just by a guideline being passed down from the Department of Health and it ends up in my pigeonhole.’ (FP15)

Training should not be seen as a process of telling practitioners what recommendations must now be followed, but a more interactive and collaborative process that recognises the autonomy of practitioners and their choice and control over deciding what is a priority for or applicable to their clinical practice. Providing evidence that the guideline has made a difference elsewhere would also be helpful. Training should involve all practitioners involved in patient care, for example, both nurses and doctors:

‘They need to hopefully be motivated in a positive way where they see it in the true spirit of quality improvement and it’s a team process and it’s for the greater good of their community that they serve. I think one cannot make it just a clinical process. There is a bit of human emotion and human motivation behind it, and there should also be a form of feedback. So I think once they buy in, they should also have say in the process of implementation, and also be involved with the feedback of each step of implementation so that they know how their own behaviour has hopefully benefitted the implementation process.’ (FP4)

Respondents thought that junior doctors and clinical nurse practitioners utilised guidelines more closely. Doctors may view the guideline as a guide to more autonomous practice, whereas CNPs may view it as a set of rules to be obeyed and strictly adhered to. In addition, guidelines could be useful for older doctors, particularly those who have not kept up to date with new developments and whose practice can be considered outdated:

‘I think a great value of the guidelines is to update people’s knowledge. I have the experience of working with colleagues who have just done their internship, and their practice is very close to the guideline, and then I work in clinics with general practitioners who have practised for 30 years, and their practice and prescription is quite different, and sometimes even dangerous, if you compare it to the guidelines.’ (FP15)

Implementation of new guidelines could be sabotaged by delay in the availability of resources. Coordination was needed between those responsible for dissemination and implementation of new guidelines with those responsible for managing the health services. Sometimes FPs might need to proactively advocate for alignment of corporate (e.g. procurement, supply chain, budgets) and clinical governance (e.g. best practice) to ensure successful implementation:

‘One of our challenges we experience is that the new guideline would come out, and say a new treatment or an intervention would be promoted, but there would be a lagging time for that medication to become available. Then the department has to adjust budgets to make it available and do this, and often there is a gap before you can actually practice what the guideline says. The inhalant corticosteroids are an example of that. It took quite a while before it became freely available, especially in the setting of our district hospital.’ (FP15)

Respondents felt that readiness to change was a huge problem in a busy and already overburdened primary care setting and different levels of readiness were reported among members of staff:

‘I think that’s an innate challenge in being human. You want to rely on what’s familiar, and it’s always challenging to change one’s own lifestyle and behaviours, even if it’s part of your professional work. Some people like to stick to what they know and what they’re comfortable with.’ (FP4)

Practitioners often find it extremely difficult to change their ways of practice, especially when they feel that control is being taken away from them or the perception remains that their current way of practising had been successful:

‘If you have been practising things for years, you’ve been taught to do things in a certain way and suddenly somebody comes and puts evidence in front of you and tells you that what you have been doing for the last 10 years has actually been harmful to your patients, or it’s not good for them, it can be very difficult for that practitioner to accept that.’ (FP7)

For many practitioners what is required may constitute a profound change in their thinking and approach to patient care and accepting the need for such a radical change may increase their reluctance to embrace the change:

‘Well, I think the paradigm shift that we sometimes have to make, because for many years we were told that this or that treatment or intervention is the best and now we have to change. I think for people who have got established practices, they often would find it very difficult to change. If they have used something that for them has worked, people are reluctant to change. So I think that is a big barrier. However, if the evidence is compelling that it doesn’t work, and then I think it is good.’ (FP2)

### Monitoring and evaluation

Respondents felt that one of the central roles of FPs was clinical governance, which entails implementing and adhering to evidence-based guidelines. There could be a tension between the desire to engage with people around implementing the guidelines and the need to audit adherence to the rules:

‘One can come up with quality improvement projects, for example, or audit. You can tell all your people in your practice that we will audit your work based on the guidelines that we have in place, because the whole idea of the guidelines is that we practise almost in a similar fashion and we use resources in a cost-effective manner.’ (FP19)

Respondents felt that quality improvement cycles work if target standards have been agreed to and owned through the guideline implementation process and the process feels reflective and appreciative rather than judgemental and critical. Good quality feedback should include feedback from patients who are on the receiving end of care. A team process was encouraged and the feedback should serve to provide ongoing motivation (not punitive action) so that ultimately practitioners are more likely to change their clinical practice:

‘So you need to have a feedback system, which is going to go in both directions, from whoever is coordinating its implementation with the staff who are going to have to implement it, with the patients as well, who are going to be the receivers of care.’ (FP7)

## Discussion

**FIGURE 1:**
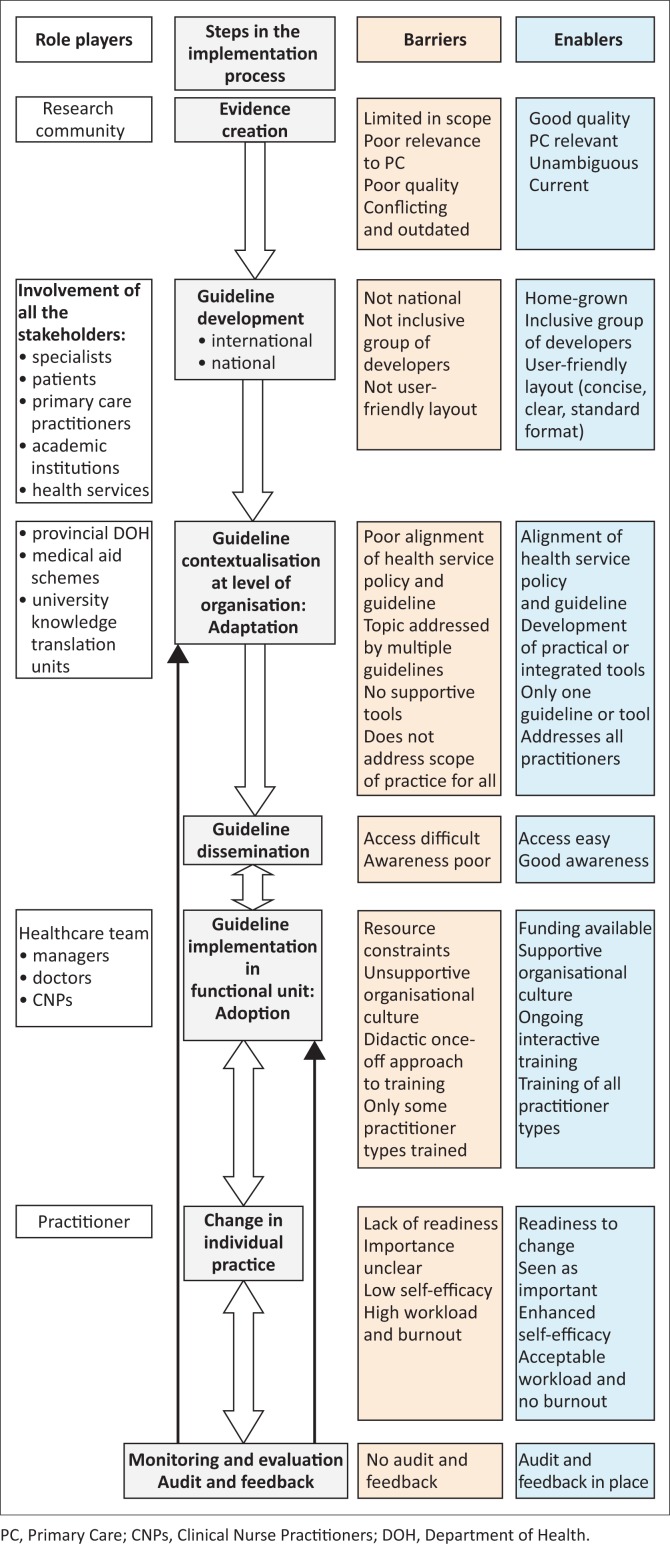
Conceptual framework.

The key findings are summarised in a conceptual framework (see Figure 1), which illustrates the key steps in guideline development, contextualisation, dissemination, implementation and evaluation as well as the interconnections between steps, and barriers or enablers to progress. Even though the conceptual framework is presented in a linear format, each of the steps involved in the guideline implementation process have challenges and complex interdependencies. The framework thus serves as a guide to action rather than a simple recipe for implementation.

Primary care researchers agree with the findings of this study that more clinical research needs to be conducted in primary care,^50^ a variety of quantitative and qualitative methods need to be embraced^51,52^ and strategies identified to engage primary care providers more in priority research.^53^

The need to formulate a more inclusive group of stakeholders in guideline development has also been recognised elsewhere and the role of patients is well recognised as they bring experiential expertise and tacit knowledge from their perspective as users of health services.^52,53,54,55,56,57^

Contextualisation of guidelines in the Western Cape Province has included an integrative process that combines individual guidelines into one decision-support tool that is disseminated further. This process has been led by a university-based knowledge translation unit. The Practical Approach to Care Kit guideline integrates primary care recommendations into algorithms for all the common presenting symptoms and chronic conditions in adults.^58^ Similar integrated tools are being prepared for children and community health workers. Integrated tools may also be able to consider comorbidity and how to balance multiple or diverse recommendations. Attention is given to the scope of practice of different primary care practitioners, such as clinical nurse practitioners and doctors, and includes all the practitioners who might be managing the condition.

There may also be a need for a more formal analysis of the target group and setting before disseminating guidelines.^59^ Information technology has created new opportunities for electronically disseminating guidelines. National primary care guidelines are now available as an application that can be downloaded onto a cell phone or tablet.^60^ Studies have also looked at the use of automated guidelines that support the decision-making process.^61^ Ensuring awareness among all relevant practitioners of a new guideline is essential for successful implementation.^62^

Dissemination alone, however, is not sufficient to ensure implementation. Availability of funding for formal implementation is often just assumed and taken for granted,^24^ and only a small number of studies report on the cost of the guideline development–dissemination–adoption process.^24^

Training on new guidelines should be interactive, collaborative and in the form of workshops rather than didactic lectures.^63^ Evidence on behaviour change should inform the design of training sessions.^64^ Implementation strategies should provide evidence for the importance of change in terms of patient outcomes.^65^

The health services should create a learning environment, encourage teamwork and have formal strategies for guideline implementation. Health services in the Cape Town metropole may lack capacity for innovation and evolution^66^ and, despite an emphasis on improving clinical processes and quality, most of this effort was experienced negatively, with staff identifying values such as not sharing information, confusion, power and hierarchy. Improving the quality of care may require transformation of the leadership style.^67^

Implementation strategies can be categorised into weak (e.g. didactic, traditional continuing medical education and mailings), moderately effective (e.g. audit and feedback) and relatively strong (reminder systems and multiple interventions).^21^ Strategies should be tailored to locally identified barriers.^68^ A number of barriers have been identified by respondents who reported time constraints, lack of financial resources, a focus on cost reduction and incongruence between corporate and clinical governance activities to be major barriers to guideline implementation in an overburdened primary care setting (Figure 1). High workload together with high levels of burnout among doctors in the Cape Town metropole^69^ may lead to resistance to commit to additional tasks. Change can also be linked to individual performance management systems and in some settings to financial incentives.^70^

Quality improvement cycles linked to guideline recommendations may be a way of reinforcing implementation as well as monitoring progress (Figure 1). Engagement of the team with the cyclical process and good quality feedback is essential. Family physicians also suggested the involvement of patients in quality improvement activities, which has been modelled elsewhere in South Africa.^71^ Monitoring and evaluation of guideline implementation may provide evidence of impact on health outcomes as well as feedback on the process of guideline contextualisation, dissemination and implementation.

Although academic FPs nationally and FPs in public and private practice were interviewed, the FPs in practice were only from the Cape Town metropole and thus are not representative of the whole country. This could limit the transferability of the findings to South Africa as a whole.

The framework of guideline development, contextualisation, dissemination, implementation and evaluation should be considered by the relevant stakeholders involved in each step of the process to guide their contribution and address likely barriers and enablers.

The study identified the need to support more primary care research in order to contribute to an appropriate evidence base for guideline development and to address the wide variety of practice-related questions with different methodological approaches.

## Conclusion

Evidence-based practice is limited in its capacity to inform primary care and a combination of qualitative and quantitative research is necessary to improve our understanding of the complexity of primary care. The research findings have been summarised in a stepwise conceptual model of guideline development, contextualisation, dissemination, implementation, monitoring and evaluation. Guideline development should be a national, more inclusive process that incorporates evidence from the primary care context. Guideline contextualisation should happen at an organisational level and may include adaptation of the guideline as well as the development of more practical or integrated tools tailored to the specific organisational context. Organisations should ensure synergy between corporate and clinical governance activities. Dissemination should ensure that all practitioners are aware of the guideline or adapted tools and know how to access them. Implementation should include training that is interactive and in a workshop format that recognises the need to address individual practitioners’ readiness to change as well as local barriers. Quality improvement cycles may reinforce implementation, monitor progress and provide feedback to the whole process of guideline implementation. Universities have a role to play with the health services in scrutinising and synthesising evidence during the development stage, translating evidence into practical tools during contextualisation and in training of staff during implementation. The proposed model may be useful for policymakers, health care managers and practitioners who are considering the use of guidelines in similar settings.
